# Self-injury: Treatment, Assessment, Recovery (STAR): online intervention for adolescent non-suicidal self-injury - study protocol for a randomized controlled trial

**DOI:** 10.1186/s13063-019-3501-6

**Published:** 2019-07-12

**Authors:** Michael Kaess, Julian Koenig, Stephanie Bauer, Markus Moessner, Gloria Fischer-Waldschmidt, Margarete Mattern, Sabine C. Herpertz, Franz Resch, Rebecca Brown, Tina In-Albon, Michael Koelch, Paul L. Plener, Christian Schmahl, Alexandra Edinger, the STAR Consortium

**Affiliations:** 1 University Hospital of Child and Adolescent Psychiatry and Psychotherapy, University of Bern, Bern, Switzerland; 2 Section for Translational Psychobiology in Child and Adolescent Psychiatry, Department of Child and Adolescent Psychiatry, Centre for Psychosocial Medicine, University Hospital Heidelberg, Heidelberg, Germany; 3 Centre for Psychotherapy Research, University Hospital Heidelberg, Heidelberg, Germany; 4 Clinic for Child and Adolescent Psychiatry, Centre for Psychosocial Medicine, University Hospital Heidelberg, Heidelberg, Germany; 5 Department of General Psychiatry, Centre for Psychosocial Medicine, University Hospital Heidelberg, Heidelberg, Germany; 6 Department of Child and Adolescent Psychiatry and Psychotherapy, University Hospital Ulm, Ulm, Germany; 7 Department of Clinical Child and Adolescent Psychology and Psychotherapy, Faculty of Psychology, University of Koblenz-Landau, Landau, Germany; 8 Department for Child and Adolescent Psychiatry and Psychotherapy, Medical School Brandenburg, Neuruppin, Germany; 9 Department of Child and Adolescent Psychiatry, Medical University of Vienna, Vienna, Austria; 10 Department of Psychosomatic Medicine and Psychotherapy, Central Institute of Mental Health, Medical Faculty Mannheim, Heidelberg University, Mannheim, Germany

**Keywords:** Non-suicidal self-injury, Adolescents, Internet, Online intervention, Randomized controlled trial

## Abstract

**Background:**

Non-suicidal self-injury (NSSI) is a clinically significant behavior affecting approximately 18% of adolescents and young adults worldwide. The importance of NSSI is supported by its association with a broad spectrum of mental disorders. Despite its high relevance, evidence-based, specific, time-, and cost-effective treatment approaches are scarce. Cognitive behavioral therapy (CBT) seems effective in reducing the frequency of NSSI in adolescents and young adults. However, young people are often reluctant to seek professional help and effective interventions adressing NSSI are not sufficiently available across all regions of Germany. Research indicates that the majority of youth with risk behavior (including NSSI) prefer technology-based interventions. To date, effective interventions for adolescents and young adults with NSSI that are deliverd online are not available.

**Methods:**

The present project aims to develop and evaluate an online intervention for adolescents and young adults with NSSI based on the content of a recently evaluated face-to-face short-term program that includes elements of CBT and dialectical behavior therapy (DBT): “The Cutting Down Programme” (CDP). The efficacy of the new online CDP intervention will be tested in a randomized controlled trial (RCT) in which *n* = 700 youths engaging in repetitive NSSI will participate in either an online psychoeducation (*n* = 350) or online CDP (n = 350). Within a postline assessment four months after baseline (end of treatment; T1), and follow-up evaluations 12 and 18 months after baseline (follow-ups; T2 and T3), NSSI and comorbid symptoms as well as quality of life will be assessed. It is hypothesized that participants receiving online CDP report a greater reduction in the frequency of NSSI within the last three months at T2 (primary endpoint) compared to those receiving online psychoeducation. Exploratory analyses will focus on predictors of treatment outcome.

**Discussion:**

We report on the development and evaluation of an online intervention for adolescents and young adults engaging in NSSI based on the CDP. If supported by empirical evidence, an online-based intervention for NSSI might help to overcome the limited availability of adequate interventions for youth.

**Trial registration:**

German Clinical Trials Register, DRKS00014623. Registered on 22 May 2018.

**Electronic supplementary material:**

The online version of this article (10.1186/s13063-019-3501-6) contains supplementary material, which is available to authorized users.

## Background

Non-suicidal self-injury (NSSI) affects approximately 17–18% of adolescents worldwide [1, 2]. NSSI is characterized by the intentional and self-inflicted destruction of body tissue without suicidal intent [3]. The importance of NSSI is highlighted by recent research illustrating that NSSI is an important predictor of suicidal behavior [4–6]. Furthermore, it is highly associated with other risk-behaviors and comorbid psychopathology [7, 8]. NSSI is a common and highly recurrent behavior that peaks in adolescence [4, 9–11].

It is essential to develop interventions supporting adolescents with NSSI to stop the behavior. However, only few interventions have been developed that specifically target NSSI despite its high clinical pertinence [12–14]. NSSI, particularly in the context of (emerging) borderline personality disorder (BPD), is often treated with Dialectical Behavior Therapy for adolescents (DBT-A), which has shown to be effective [15]. Other treatments that seem effective for reducing NSSI frequency are Mentalization-Based Treatment for adolescents (MBT-A) as well as Cognitive Behavioral Therapy (CBT) [16, 17]. A recent meta-analysis [16] suggested that DBT, MBT, and CBT may all be effective for treating NSSI. However, comprehensive access to these treatments is restricted due to limited resources and a lack of specially trained clinicians [18]. Given the limited resources in the mental healthcare field, it remains a matter of discussion if DBT-A and MBT-A, which are targeted at youth with BPD need to be fully implied.

Thus, less intensive yet effective programs that focus specifically on NSSI are needed to improve the general standard of care for affected individuals [19], particularly for adolescents reluctant to seek professional help despite their actual need. Indeed, help-seeking in adolescents engaging in NSSI is considerably low [20–22]. Adolescents endorsing NSSI express more negative attitudes towards help-seeking compared to past self-injurers and adolescents with no history of NSSI [22]. Negative attitudes towards help-seeking in adolescents and young adults with NSSI frequently arise from the disclosure and related stigma of the behavior towards parents, peers, and mental health professionals [23]. Typically, adolescents engaging in NSSI turn to the Internet in order to find peer support [24]. Although such exchange may actually foster NSSI, by reinforcement through the sharing of stories and strategies of NSSI [25], it also offers opportunities for therapeutic interventions [26]. Studies [27, 28] have shown that a significant percentage of adolescents and young adults (45–93%) with risk behavior (including NSSI) prefer a technology-based intervention format (versus an in-person face-to-face intervention). Such youth-adequate delivery of a NSSI intervention may lower barriers towards care and reach adolescents and young adults in rural areas, where adequate help is not sufficiently available. To date, the access to effective intervention requires great efforts and waiting times, especially for adolescents and young adults in rural regions. We have previously shown that the time that adolescents need to travel to receive professional help for their mental health problems hinders actual help-seeking behavior [29]. Online-based interventions could overcome these barriers.

However, to date, no effective online-based intervention for adolescents engaging in NSSI exists. Time- and cost-effective brief interventions exclusively targeting NSSI are rare. Therefore, we have developed an online intervention for adolescents and young adults with NSSI based on the content and our experience with a cognitive-behavioral short-term program for adolescents engaging in NSSI. In 1999, a short-term psychotherapy was developed for adults exhibiting deliberate self-harm, the manual-assisted cognitive-behavior therapy (MACT) [30]. In 2011, the MACT was adapted for adolescents within “The Cutting Down Programme” (CDP) [31], consisting of 8–12 sessions. Our group has just completed the first randomized controlled trial (RCT) on the effectiveness of face-to-face CDP [19] including *n* = 74 adolescents engaging in NSSI. Findings are promising and are currently under review.

To deliver such effective interventions online may increase the availability and offer the opportunity to provide adolescents and young adults concerned with adequate intervention [32]. Concerning the low help-seeking behavior within the particular target group, the low threshold access, low stigma, and high confidentiality offered by online interventions are promising in reducing the treatment gap. Consequently, our proposed online intervention could lower barriers for a high-risk target group to seek professional help.

### Objectives and outcomes

The main objective of the present study is to evaluate a newly developed, easily accessible, online intervention program for adolescents and young adults engaging in NSSI, based on an existing short-term face-to-face manualized intervention (CDP). The effectiveness of online CDP will be evaluated in a RCT in comparison to an online psychoeducation program. The main outcome of the trial is the reduction in the frequency of NSSI within the last three months assessed by the NSSI Severity Questionnaire (NSSV-SG) [33] at one-year follow-up (primary endpoint; T2).

Secondary outcome criteria are health-realted quality of life as well as comorbid psychopathology and suicidal behavior.

### Primary hypothesis

Participants receiving online CDP will show a significantly greater reduction in the frequency of NSSI within the past three months at T2 compared to participants within the online psychoeducation group.

## Methods/Design

### Setting and recruitment

The present trial is a study within the consortium “Self-injury-Treatment: Assessment, Recovery (STAR).” Cooperating study sites (Ulm, Landau, Mannheim, Karlsruhe, and Berlin) will investigate the natural course of NSSI as well as psychocological and neurobiological predictors and improve ways to dessiminate specific knowledge on NSSI to primary caregivers.

**Fig. 1:**
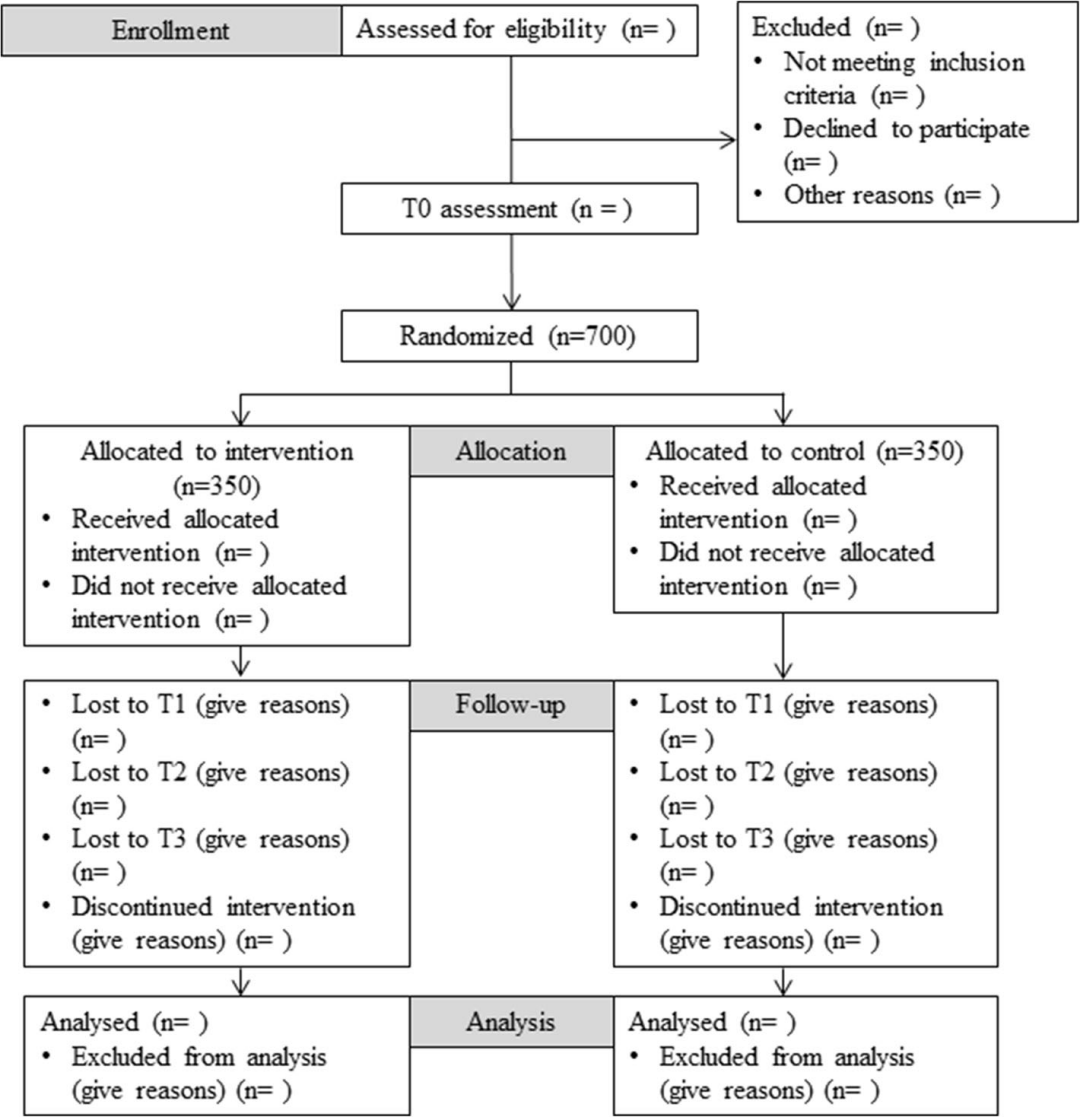
Consolidated Standards of Reporting Trials (CONSORT) 2010 *flow diagram*

**Fig. 2:**
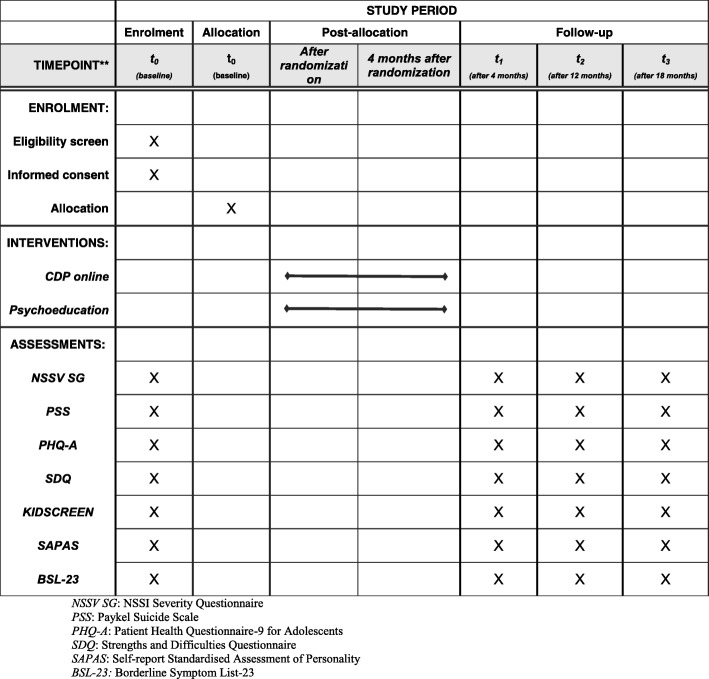
SPIRIT figure: Schedule of enrollment, interventions, and assessments

We aim to include *n* = 700 adolescents and young adults aged 15–21 years in the trial. They will be randomly assigned to either receive the online CDP or online psychoeducation only. Participants in both groups will be assessed at the beginning (baseline; T0), 4 (post-line; T1), 12, and 18 months after the initial baseline assessment (follow-up; T2, T3) in order to evaluate treatment effects. The flowchart of the trial is shown in Fig. 1. The schedule of this trial is shown in Fig. 2 (Additional file 1).

Recruitment is conducted online through social media (such as Instagram and Facebook), websites, and online forums. Furthermore, flyers will be printed and disseminated at the clinical centers participating in the study (Berlin/Neuruppin, Heidelberg, Landau, Mannheim, Ulm). All eligible participants registering on the central project website will be offered to participate in the online intervention.

### Inclusion and exclusion criteria

Based on the criterion A of the NSSI disorder provided in the *Diagnostic and Statistical Manual of Mental Disorders* (DSM), fifth edition, section three [3], eligible participants are required to have engaged in NSSI on at least five days during the last 12 months. Eligible participants are aged 15–21 years. Furthermore, all participants need to provide informed consent online by clicking a checkbox. Participants will be excluded if they are currently receiving individual psychotherapy (inpatient and outpatient).

### Procedure and randomization

Before inclusion, participants will be provided with written information on the study’s background, aims and procedures, the online CDP and online psychoeducation, randomization, the advantages and risks of participating, data collection and data safety regulations, as well as voluntary participation.

Informed consent will be obtained after an initial screening phase within T0 to check for eligibility and before registering on the study website. Participants confirm by ticking a box on the website that they have read and understood the provided information and that they are willing to participate.

Within the self-report assessment and on the study platform, participants will be informed that there is no immediate check of the data they enter and thus no immediate measures can be guaranteed in case a participant reports acute suicidality. Participants will be made aware of emergency numbers in case of acute suicidality.

After providing informed consent, participants have to provide an email address as well as a username in order to register for the study. An activation link will be sent to the email address provided. Once the address has been verified, a unique code will be generated and assigned to the participant, in order to enable the matching of follow-up assessments.

After consent and registration to the platform, all eligible participants will be asked to complete a 45-min assessment within T0. After completion of the assessment at T0, participants will be randomized to the online CDP or the online psychoeducation and will be invited to participate in assessments at T1, T2, and T3. To promote participant retention, participants will be reminded to participate in the assessments via standardized e-mails at each point in time. Within a first e-mail, participants receive an invitation as well as a link to the assessment. After 3 and 14 days, participants receive reminder e-mails for the assessment. This applies to every assessment point.

To prevent bias, participants will be randomly assigned to receive either the online psychoeducation or the online CDP using block randomization (50/50). Participants will be randomized based on their study ID. Enrolment, generation of allocation sequence, and assignment of participants take place online and are therefore computer-based. Participants will be informed about their group affiliation by automatic message that pops up immediately after randomization. Blinding is not completely possible. Participants cannot be blinded due to the different nature of the interventions. Blinding of the researchers is not possible, because participants in the online CDP group receive case-management and personal chats as well as telephone calls.

### Data assessment

All assessments will be conducted online. Thus, self-report questionnaires will be used in this trial. The assessment at T0 takes place before randomization and the subsequent start of treatment. All assessment measures are conducted at every time point (T0, T1, T2, T3), except the evaluation of the online CDP, which only takes place at T1. Within a first screening phase of T0, participants are checked for eligibility. All eligible participants are invited to participate in the trial. Those not eligible for the trial because of current individual psychotherapy and/or too low frequency of NSSI are invited to participate in a separate non-interventional observational study.

### Assessment measures

T0 comprises sociodemographic questions, which will gather information about gender, nationality, state of adolescents’ and parents’ education, and current living situation.

For detailed assessment of NSSI severity, we use the NSSV-SG [33], assessing the frequency and nature of NSSI within the past three months using 60 items. The evaluation of the psychometric properties in a clinical and an online sample of adolescents and young adults indicates high internal consistency (Cronbach α = 0.73), a test–retest reliability of 0.77 (*n* = 29; interval: six weeks), and supports the validity of the questionnaire [34]. The *KIDSCREEN-10* will be used to assess general health-related quality of life (HRQoL) in participants [35]. It consists of 10 items and was developed across Europe for use in children aged 8–18 years and is a valid instrument with acceptable retest reliability [35]. Internal consistency was high (Cronbach α = 0.81) [36]. To assess emotional and behavioral difficulties in children and adolescents, the *Strengths and Difficulties Questionnaire* (SDQ) [37] with 25 items will be used. A study, evaluating the validity of the German version of the SDQ, indicates that the SDQ is valid for most clinical and research purposes [38]. In addition, the questionnaire shows good internal consistency (Cronbach α = 0.81) [39]. The *Borderline Symptom List-23* (BSL-23) [40] is a quantitative assessment of borderline-specific symptoms based on DSM-IV criteria [41]. It consists of 23 items and shows high internal consistency (Cronbach α = 0.94) [40]. The BSL-23 is a reliable and valid instrument to assess borderline-specific symptoms [40]. To assess personality disorders, the *Self-report Standardised Assessment of Personality* – abbreviated scale (SAPAS-SF) consisting of eight items [42] will be used. The SAPAS is a valid and reliable instrument to screen for personality disorders [42, 43]. The internal consistency coefficients were rather low, in the range of 0.35–0.51 [44]. The *Paykel Suicide Scale* (PSS) [45] assesses the intensity of suicidal behaviors by level of intent and consists of five items. The *Patient Health Questionnaire-9 for Adolescents* (PHQ-A) [46] is a modified version of the *Patient Health Questionnaire-9* (PHQ-9) [47]. The PHQ-9 measures depression severity and shows good internal consistency (Cronbach α = 0.84) [48]. The PHQ-A consists of nine items based on DSM-IV [41]. The PHQ-A is a reliable and valid measure of depression severity [46] and shows good.

Beyond that, additional items will be presented to evaluate the online CDP. Within 20 items, participants will rate how helpful the different sessions have been and what they have learned on a 5- and 6-point Likert Scale, respectively. Furthermore, the revised short form of the Working Alliance Inventory (WAI-SR) [49] consisting of 12 items was used to assess the therapeutic relationship. Internal consistency was excellent (Cronbach α > 0.90) [50]. Finally, a modified version of *The Fragebogen zur Patientenzufriedenheit* (ZUF-8; Patient Satisfaction Questionnaire) was used to assess treatment satisfaction. It consists of eight statements, which are answered on a 4-point scale ranging from ‘very satisfied’ to ‘quite dissatisfied’ [51].

### Participants’ incentive

Participants receive no direct financial compensation for participating in the study. However, they are informed about the chance to win a gift voucher (€50) for their participation. After completion of T1 assessment, participants have the chance to win the voucher.

### Interventions

*N* = 700 participants will be randomized to one of the two possible treatment conditions (online CDP versus online psychoeducation). Table 1 provides an overview of the interventions in both groups. The platform is available for four months for each participant after randomization. After a successful evaluation of the online CDP, the intervention could be disseminated to provide all individuals concerned with access to the online CDP. However, there are no plans concerning an immediate access to the intervention for the control group after conclusion of the trial.

**Table 1:** Overview of interventions

Group affiliation	Intervention
Both groups	Online psychoeducation:
	- basic facts on NSSI, NSSI and emotions, NSSI and development, treatment for NSSI in terms of plain information
	- static content
	- dose of the psychoeducative intervention is determined by the user
	- access for four months
Control group	Online psychoeducation
Intervention group	Online psychoeducation
	Monitoring module:
	- weekly assessments on NSSI and the use of skills for individual feedback
	Add-on online CDP:
	- access to 10 different sessions: (1) identifying resons for NSSI, getting to know skills; (2) promoting motivation; (3) getting to know and dealing with feelings; (4) establishing positive activities; (5) understanding the links between thoughts, feelings, and behaviors and learning how to find a more helpful way of thinking; (6) identifying core beliefs and rules of living; (7) identifying coping strategies; (8) promoting assertiveness; (9) identifying strategies and skills that are based on the concepts of mindfulness and distress tolerance; (10) exploring triggers for NSSI, reviewing coping strategies - techniques: chat and telephone calls with case managers, moderated group chat, exercises, videos, quizzes - access for four months

### Online psychoeducation

The online psychoeducation provides static psychoeducative content on the causes, consequences, and concomitants of NSSI. This module is available for both groups. Recommendations concerning the intensity of use are rather individual depending on the already existing knowledge on NSSI. Thus, the intensity of use is determined by the participant and tracked as a variable of interest.

### Online CDP

The online CDP group is offered an add-on online intervention. The face-to-face, short-term intervention is based on elements of CBT and DBT and is specifically tailored for the treatment of NSSI in adolescents. The treatment length is 8–12 sessions. The treatment consists of four modules that can be expanded by optional exercises. Module 1 focuses on providing knowledge about CBT and NSSI as well as promoting therapy motivation. The focus of module 2 is on identifying the reasons for the NSSI. In module 3, the patients are encouraged to test alternative behaviors to NSSI and module 4 comprises the stabilization of the alternative behaviors. The content of the intervention is structured in a manual for participants and a separate manual for therapists that is feasible for translation into an online intervention. The modules in the manual have all been developed based on a comprehensive literature review on NSSI including associated psychological phenomena. Within the online CDP, the intervention is delivered via personal chat or telephone calls with the case managers and assisted by automatic content of the web-based platform, which provides 10 different sessions. The sessions comprise content of the face-to-face manual adapted for online use. In addition to text, participants are provided with exercises and quizzes to deepen their theoretical knowledge on the one hand and to ensure understanding of the provided content on the other hand. In addition, videos of two fictitious patients are provided in every session. These fictitious patients present common problems and how they deal with them. The online CDP group will further have access to a moderated online group chat facilitating exchange with other participants and providing peer support. Additionally, all participants in the online CDP group are monitored for NSSI and the use of skills on a weekly basis to receive an individual feedback on their progress. The intervention will solely be provided over the Internet.

Participants are encouraged to use the online CDP as often as they want to. There is no restriction or specification concerning the intensity of use. Again, the use of the online CDP is determined by the individual. Individual contacts with the case managers via personal chat or telephone call are regularly offered once a week. However, further appointments are possible if particular circumstances require additional support. Group chats also take place once a week.

All participants are permitted to seek treatment outside of the clinical trial if necessary. The nature and frequency/dose of interventions outside of the trial will be assessed using structured questionnaires.

#### Emergency procedure

If participants within the online CDP group indicate serious suicide thoughts within a chat or telephone call with a case manager, suicidality is clarified by the respective case manager in a first step, as well as by the responsible psychiatrist, as needed. Participants will then be referred to emergency numbers.

There are no general discontinuation criteria for the trial.

### Staff

All case managers are clinical psychologists who receive a priori training in the online CDP intervention. Furthermore, case managers will receive weekly supervision by a specifically trained clinical psychologist with a clinical background in CBT and DBT to ensure the quality of treatment. In addition, monthly external supervision will be offered by a specifically trained psychotherapist in CBT and DBT. Beyond that, psychiatrists both for children and adolescents as well as for adults who also receive a priori training in the online CDP are available in cases of emergency. All case managers are based in Heidelberg.

### Sample size

Power analysis was based on reported effect sizes of CBT in the reduction of NSSI, drawing on the most extensive meta-analysis in the field [52] reporting a main effect for the reduction of NSSI in *n* = 8/14 studies; g = − 0.27, 95% CI [− 0.17, 0.38], z = 4.96, *p* = 0.001). Most studies included in the analysis used a treatment as usual (TAU) control group design. Comparing online CDP against an online psychoeducation we expect similar effects. Power analysis was calculated for F-statistics using an alpha-error probability of 0.05. The estimation model was based on a mixed effect linear regression with main effects (repeated measures and group) and their interaction. We considered covariates with potential impact on the outcome (sex, age). Based on the calculation, a minimum sample size of *n* = 344 is necessary to reveal significant effects at a critical F-value of F = 1.859. Based on this estimation, the study would allow for up to 51% drop-out and still be sufficiently powered revealing effects in a completer analysis.

### Statistical analysis

The primary hypothesis will be addressed using a mixed linear regression analysis with robust variance estimation, assessing the frequency of NSSI within the past 3 months at T0 and T2. We will consider main and interaction effects of the two main effects: (1) group allocation; and (2) time of assessment within repeated measures. The individuals’ ID will be used as random effect variable. In secondary analyses, potential sociodemographic and clinical mediators and predictors of intervention response will be investigated. Missing data and individuals withdrawn from the trial will be handled using an intention-to-treat (ITT) approach. All participants randomized will be considered in the analyses. In the case of drop-outs or missing data, we will use a last-value-carried-forward imputation method, assuming that those who dropped out of the intervention showed no improvement in NSSI relative to baseline.

### Data safety

The confidentiality of research participants is secured by providing unique study identifiers unrelated to the real name.

The Coordination Centre for Clinical Trials (KKS) in Heidelberg is involved in preparing the investigator site file. Due to the online-based nature of the intervention, classical site visits are not necessary. Instead, pre-trial visits in terms of profound explanation of online content and all aspects related to delivery of the intervention took place. In addition, an independent data safety and monitoring board (DSMB) was established to monitor the collection and analyses of data.

Computerized assessments guarantee the highest level of data integrity and quality, i.e. missing data will be minimized and false data entry will be prevented. Online access allows for continuous monitoring of data collection, documentation of access logs, and traceability of all entered data (user and timestamp) as well as restoration of all previous states. A Distributed Replicated Block Device (DRBD)-based cluster will provide synchronous replication of all data during data entry on two separate servers and highest availability. In addition, full and incremental backups will be conducted following a predefined back-up plan. Data storage and transfer will be encrypted. Access to the data will be strictly limited to authorized persons and will be password-protected. All servers are located at the University Hospital Heidelberg.

### Harms

In view of the non-invasive intervention, the risk for participants is considered marginal. Irrespective of the individual allocation to one of the treatment arms, participating adolescents and young adults benefit from receiving psychoeducation on NSSI and associated mental distress. Adolescents will be instructed on the crisis procedures mentioned above. There is no obvious risk for participants. The independent KSS Heidelberg will provide expert advice and monitoring regarding all aspects of safety. Beyond that, the DSMB will be informed about adverse events, such as suicidal acts.

### Ethical issues and dissemination

The study will be conducted in accordance to the declaration of Helsinki and the rules for physicians of the medical association (“Landesärztekammer”) of Baden-Württemberg in their currently valid version. Study participation is voluntary. Consent can be withdrawn at any time without stating the reason and without any individual disadvantage for subsequent medical care. All participants will need to consent electronically to the terms of the study. In case of study withdrawal, previously collected data will be destroyed if desired unless data are already included in analyses. A waiver for parental consent was obtained from the institutional review board of the medical faculty at the University of Heidelberg. Ethics committees are able to waive paternal informed consent if studies are expected to result in great benefits for a specific target group or if studies could not be conducted otherwise [53]. Both points apply to the present study. Beyond that, the study is a non-invasive low-risk study and within the medical community, the age of 14 is considered to be the age when minor participants are able to consent to participation in low-risk studies [54, 55]. Cognitive abilities are comparable to those in adults at the age of 14 years [56]. Furthermore, the study is about reducing obstacles in receiving professional help. Thus, asking for parental informed consent would increase hurdles, especially for adolescents who are often struggling to receive adequate professional help [57].

The study protocol has been approved by the ethics committee in Heidelberg for consulting of professional conduct before the start of our study.

In the case of relevant protocol modifications, the institutional review board of the medical faculty at the University of Heidelberg will be informed and an amendment will be submitted. Furthermore, information within the German Clinical Trials Register will be updated to inform the public about possible changes.

All confidential information is subject to medical confidentiality and to the requirements of the European, Federal and State Data Protection Act (Europäische Datenschutzgrundverordnung, EU-DSGVO, Bundesdatenschutzgesetz, BDSG and Landesdatenschutzgesetz, LDSG). The data will be stored and processed in a pseudonymized manner. No third parties will gain insights into the original data.

Beyond regular journal publication, general strategies are planned to disseminate trial results: coordination and participation in scientific meetings (scientific dissemination) as well as dissemination of materials created in the subproject STAR-TRAIN, which develops practical instructions for the contact to adolescents and young adults with NSSI in primary care practice (dissemination to the public). The use of professional writers is not intended. Access to the protocol is ensured through the registration and regular update of the trial in the German Clinical Trials Register (STAR: Self-Injury: Treatment, Assessment, Recovery - Online Intervention for Adolescent Nonsuicidal Self-Injury - A randomized controlled trial; http://www.drks.de; DRKS00014623; registration date: 22 May 2018).

## Discussion

NSSI is a significant problem in adolescents and young adults, associated with severe psychopathological distress and potential long-term consequences [4–7]. Available intervention options are limited in their outreach and often fail to reach youth in rural areas and those with low help-seeking behavior. An online intervention may help to overcome existing barriers and improve general access to care for adolescents and young adults engaging in NSSI. However, so far, no study has addressed the effectiveness of online interventions for this target group. Thus, this is the first RCT to develop and evaluate an Internet-based short-term treatment for NSSI in adolescence and young adulthood. The present study and related intervention make several advances in the treatment of NSSI and build upon previous work in innovative ways.

First, we focus on NSSI, which is a significant issue in the mentioned age group. Second, we provide an evaluated short-term program addressing NSSI specifically. Third, we use an Internet-based approach to deliver therapy content. Intervening via this medium offers a number of advantages over face-to-face interventions and promises to reach a significant number of affected individuals regardless of their place of residence. In addition, help-seeking is considerably low in the target group and online interventions may lower barriers for young people to seek help.

## Limitations

Despite the mentioned advantages, potential limitations of the study design should be acknowledged. Participant drop-out is a potential challenge, particularly given the online nature of the intervention. Based on a Cochrane Review [58], attrition rates from 4% [59] up to 21% [60] were reported within face-to-face treatments of adolescent self-harm (including NSSI). Concerning online interventions for adolescents with diagnoses of depression [61, 62] and anxiety [61, 63], drop-out rates from 6% [64] up to 31% [65] were reported. Considering the mentioned studies, an attrition rate of 30% to T1 and 50% to T2 is expected, expecting *n* = 504 adolescents to complete the intervention. The study is suitably powered even down to a completer sample of *n* = 344 adolescents.

Another potential limitation is that all participants will be able to access alternative treatments outside of the trial. Information will be collected from participants about menthal health service usage and the potential impact of this on outcomes will be statistically controlled for.

In addition, we are not able to diagnose mental disorders because of the exclusive use of surveys. This is due to the online character of the study.

The provision of an online intervention, tailored to the needs of adolescents and young adults, may provide an easily accessible, cost-effective, and flexible medium for improving mental health outcomes for affected individuals.

## Trial status

Protocol version 1.0, 25 April 2018.

The trial is scheduled to be completed by 31 October 2021. Recruitment began on 1 November 2018 and recruitment will be completed approximately by 31 October 2020.

## Additional file

Additional file 1: SPIRIT 2013 Checklist: Recommended items to address in a clinical trial protocol and related documents*. (DOC 123 kb)

## Data Availability

Not applicable.

## Abbreviations

BDSG: Bundesdatenschutzgesetz
BPD: Borderline Personality Disorder
BSL-23: Borderline Symptom List-23
CBT: Cognitive Behavioral Therapy
CDP: Cutting Down Programme
DBT-A: Dialectical Behavior Therapy for Adolescents
DRBD: Distributed Replicated Block Device
DSM-5: Diagnostic and Statistical Manual of Mental Disorders, Fifth edition
EU-DSGVO: Europäische Datenschutzgrundverordnung
ITT: Intention-to-treat
KIDSCREEN: Health-Related Quality of Life Questionnaire for Children and Young People
KKS: Coordination Centre for Clinical Trials
LDSG: Landesdatenschutzgesetz
MACT: Manual-assisted Cognitive Behavior Therapy
MBT-A: Mentalization-Based Treatment for Adolescents
NSSI: Non-suicidal self-injury
NSSV-SG: NSSI Severity Questionnaire
PHQ-A: Patient Health Questionnaire-9 for Adolescents
PSS: Paykel Suicide Scale
RCT: Randomized controlled trial
SAPAS-SF: Self-report Standardized Assessment of Personality – abbreviated scale
SDQ: Strengths and Difficulties Questionnaire
TAU: Treatment as usual
